# Quality of life after lung cancer surgery: sublobar resection versus lobectomy

**DOI:** 10.1186/s12893-023-02259-1

**Published:** 2023-11-18

**Authors:** Shuai Jiang, Bao Wang, Mengzhe Zhang, Zuo Liu, Zengtuan Xiao, Jialin Gong, Xiaofei Wang, Zhenning Zhang, Zhenfa Zhang

**Affiliations:** Department of Lung Cancer Surgery, Tianjin Medical University Cancer Institute and Hospital, Tianjin Medical University, 1 Huanhu West Road, Tianjin, 300020 China

**Keywords:** Non–small–cell lung cancer, Postoperative quality of life, Sublobar resection, Lobectomy

## Abstract

**Background:**

This study aimed to compare the postoperative quality of life (PQOL) between non–small–cell lung cancer (NSCLC) patients who underwent video–assisted thoracoscopic sublobar resection (subsegment, segment, or wedge) and lobectomy. Meanwhile, we developed a PQOL scale for patients with NSCLC after optimization.

**Methods:**

Developing and evaluating the postoperative quality–of–life scale of non–small–cell lung cancer (NSCLC–PQOL) followed by the international principles for developing quality–of–life scale. Therefore, we used the NSCLC–PQOL scale to evaluate the PQOL of patients who underwent different surgeries.

**Results:**

The overall PQOL of patients who underwent video-assisted thoracoscopic lobectomy and sublobar resection gradually worsened from discharge to 3 months postoperatively and progressively improved from three to 6 months postoperatively. And the sublobar resection group showed better PQOL in chest tightness, breath shortness, breathlessness, cough and expectoration than the lobectomy group, and the differences were statistically significant (*P* < 0.05). The final version of the NSCLC–PQOL contained three dimensions: “signs–symptoms”, “psychological and psychiatric”, and “social–life” dimensions.

**Conclusions:**

The sublobar resection group showed better PQOL in “chest tightness”, “breath shortness”, “breathlessness”, “cough”, and “expectoration” than the lobectomy group. Twenty–two items formed a well–behaved PQOL scale after being validated satisfactorily. The scale was a suitable rating tool for evaluating the NSCLC–PQOL of patients.

**Trial registration:**

As this study was a retrospective study and not a clinical trial, we did not register this study in the Chinese Clinical Trial Registry.

**Supplementary Information:**

The online version contains supplementary material available at 10.1186/s12893-023-02259-1.

## Introduction

### Background

Lung cancer is one of the malignant tumours associated with high incidence and mortality rates, which accounts for 11.4% of all cancers. Among them, NSCLC accounts for 80–85% of lung cancer [1, 2]. Based on earlier research, lobectomy has been the standard surgical treatment for NSCLC [3]. With the pulmonary nodules being detected more frequently, benefiting from the introduction of lung cancer screening, we could preserve lung function for such patients by receiving sublobar resection at an early stage. Sublobar resection with selective or no lymph node dissection may be sufficient for patients with small ground glass opacity (GGO) lesions, especially GGO dominant nodules [4]. But there has been no standard operation for such patients. It is well known that the core of the treatment is survival. Results of a meta–analysis in Japan revealed that the postoperative survival showed no significant difference between limited resection (wedge resection or segmentectomy) and lobectomy for Early–stage (stages I) NSCLC, the differences between one–year, three–year and five–year survival rates of two groups were 0.7, 1.9 and 3.6%, and the differences were not statistically significant [5]. Another study also showed that the 10–year survival between the wedge resection and lobectomy groups had no difference [6].

Meanwhile, there was no significant difference in overall survival (OS) among patients who underwent lobectomy and segmentectomy [7]. Thus, while performing complete resection with negative margins and biopsies of the adequate number of lymph nodes (LNs), there was no difference between patients who underwent sublobar resection and lobectomy at an early stage.

As the development of examination methods and treatment results in better prognosis, clinicians are focusing more on the PQOL of patients. As dyspnea, cough, fatigue and insomnia could be interrelated symptoms after surgery [8], the PQOL of patients could be comprehensively evaluated through the associated symptoms and social and psychological factors. Thus, the PQOL becomes an important indicator for evaluating the advantages of the two surgical procedures. But there is no gold-standard instrument to evaluate PQOL for NSCLC patients. Functional assessment of cancer therapy–lung (FACT–L) scale has too many items, which leads to poor adherence; The lung cancer symptom scale (LCSS) can’t reflect the multidimensionality of PQOL; Meanwhile, the short form 36 health survey questionnaire (SF–36 Health Survey) isn’t a pulmonary–specific quality of Life (QOL) Scale. While applying the FACT–L scale entries, the researcher will directly ask the respondents questions related to life and death that are too aggressive [9]. Therefore, it may lead to poor compliance by the respondents. Besides, the LCSS scale does not provide an in–depth assessment of the quality of existence related to the area of social life [10], due to the stated goal of the LCSS scale is the symptom control evaluation. At the same time the SF–36 is not a quality of life scale for patients with specific diseases, which may lead to inappropriate evaluation of QOL during the evaluation process [11]. This study aimed to evaluate whether there was any difference in PQOL among patients who underwent lobectomy and sublobectomy. Meanwhile, we developed a more comprehensive PQOL scale for patients with NSCLC with shorter finishing times.

## Methods

### The development of the scale

#### Launching the study group

The Experts Reference Group (ERG) comprises thoracic specialists, nurses and researchers from Tianjin Medical University Cancer Institute and Hospital. The core group members are postgraduates. They collected and organized initial entries and then gave out clinical questionnaires. After that, they finally collected and statistically analyzed data. Before undergoing clinical investigations, we trained investigators, consisting of students from Tianjin Medical University.

#### Developing the framework of the scale

We followed the guidance of international regulation to develop the scales [12, 13]. We finally determined the three-tier scale structure and then developed three dimensions of the QOL scale spanning the signs and symptoms subscale, psychological and psychiatric subscale and social life subscale.

#### Building the initial entry

(1) Consulting and analyzing the literature: We developed the scale framework after combining FACT–L, LCSS and SF–36 Health Survey. (2) Case analysis and clinical investigation: Consecutive NSCLC patients who visited the department of Lung Tumor Surgery of Tianjin Medical University Cancer Institute and Hospital from September 2019 to May 2021 were retrospectively investigated. Postoperative follow–up was achieved via phone call. (3) Consulting Experts: ERG members respectively proposed entries which could influence patients’ PQOL. Then the collection and curation were finished by core group members.

#### Preliminary investigation and development of initial scale

(1) Setting response options: We set the answer with a visual simulation scoring method (e.g., the assignments of responses for the questions ranging from “not at all” to “very much” are equal to “0” points to “3” points). (2) Preliminary investigation: We randomly followed up with three NSCLC patients and 10 healthy people over the phone. All of them reported that the scale had no ambiguity.

#### Clinical investigation and development of final scale

We screened the entries after clinical investigation and statistical analysis. Then, the data was collected over the phone. (1) The study subjects and manners of the survey: The study enrolled 347 patients who visited the department of Lung Tumor Surgery of Tianjin Medical University Cancer Institute and Hospital from September 2019 to May 2021, meeting the inclusion criteria. All patients signed consent form for surgery before surgery. The study was approved by the Ethics Committees of the Tianjin Medical University Cancer Institute and Hospital (ethics ID bc2021134) and was conducted in accordance with the national guide-lines and the Declaration of Helsinki. A relevant study showed that lung cancer surgery might cause further deterioration of QOL, especially in the first 3 to 6 months after surgery [14]. Therefore, we conducted follow–up data collection via phone call at hospital discharge level, 3 and 6 months after surgery. (2) Inclusion criteria: ① Patients aged from 18 to 80 years old; ② Patients who underwent video-assisted thoracoscopic sublobar resection, lobectomy, wedge resection or combined–subsegments resection; ③ All patients were diagnosed with NSCLC by pathology or cytology; (3) Exclusion criteria: ① Patients who received antitussive drug therapy within 2 weeks before surgery; ② Patients converted to open thoracotomy or underwent secondary surgery; ③ Patients who underwent pneumectomy; ④ Patients complicated with asthma, bronchiectasis, pulmonary tuberculosis and severe cough; ⑤ Patients who were dead; ⑥ Patients underwent multiple surgeries or both sublobar resection and lobectomy; ⑦ Patients with impairments that significantly limited their ability to communicate or understand; ⑧ Patients with serious primary diseases; ⑨ Patients who had a history of mental illness or were unable to cooperate with questionnaires; (4) Developing and distributing clinical questionnaires: The data was collected through phone calls by using the primary NSCLC–PQOL scale. (5) Clinical survey and quality control: While filling out the questionnaires, investigators could properly explain the entries when patients were confused. After the survey, the database was established by *Microsoft Excel 2010*, and all questionnaires were entered twice by different data entry personnel. Raw data was retained, and two investigators audited each other’s data for accuracy. Then the analysis was carried out by *SPSS 26.0*. (6) Screening items of the scale by statistical methods: The research group screened the initial scale items by five internationally recognized methods of items distribution method, discrete tendency method, correlation coefficient method, Cronbach’s α coefficient method, and multiple stepwise linear regression method. On this basis, the central tendency, internal consistency, representativeness and other properties of the data were analyzed, and the items unsuitable for the final scale were eliminated to form NSCLC–PQOL scale.

#### Evaluation of the final scale

The evaluation of the scale includes internal consistency, content validity, structural validity and other characteristics [15].

### Univariate analysis

Quality of survival scores at 3 and 6 months postoperatively were used as the dependent variable, smoking history, pathological staging, imaging characteristics, sex, age, and TNM stage were used as independent variables. If the independent variables were dichotomous, then analyzed data with the Mann–Whitney U test. If independent variables were multi categorical variables, then analyzed data with the Kruskal–Wallis test.

### Multifactorial analysis

Multiple linear regression analyses were performed with quality of survival scores at 3 and 6 months postoperatively as the dependent variable and smoking history, pathological staging, imaging characteristics, gender, age, and TNM stage as the independent variables.

### Comparison and analysis of quality of survival

Median, mean, and standard deviation were calculated for descriptive data; rank sum test was used for hierarchical data and non-normally distributed data.

### Evaluation and comparison of NSCLC–PQOL

We conducted follow–up data collection via phone call at the hospital discharge level, 3 and 6 months after surgery. For a single item, we used the Mann-Whitney U test to compare the PQOL between the two groups. And for the comparison of the total scale score between the two groups, we used the independent samples t–test.

## Results

### Development and evaluation of the scale

The process of establishing and evaluating the NSCLC–PQOL scale is described in the Supplement materials, and the NSCLC–PQOL scale has been evaluated and qualified with good reliability, validity, and feasibility, and can be used as a tool for evaluating the quality of survival of patients with non–small cell lung cancer.

### Application of scale scoring

As the baseline levels of patient demographic characteristics are not consistent (Table 1), we first conducted univariate and multivariate analyses to exclude confounding factors. A univariate analysis was performed with the postoperative quality of survival scores at 3 and 6 months as the dependent variable, and smoking history, pathological staging, imaging characteristics, gender, age, TNM stage, surgical procedure as the independent variables to exclude the interfering factors. The analysis showed that apart from the surgical procedure, only the TNM stage affected the quality of survival at 3 months postoperatively (Table 2). Further onwards, we proceeded to include smoking history, pathological staging, imaging characteristics, gender, age, and TNM staging in the multiple linear regression analyses with the quality of postoperative survival score as the dependent variable at 3 and 6 months, respectively. At 3 months after surgery: the regression model was not statistically significant (*P* > 0.05), F = 1.74, *P* = 0.13, adjusted R^2^ = 0.01, suggesting that factors included in the model other than surgical modality did not affect the quality of survival. At 6 months after surgery: the regression model was not statistically significant (*P* > 0.05), F = 0.93, *P* = 0.46, adjusted R^2^ = −.001, suggesting that factors included in the model other than surgical procedure did not affect survival quality.

**Table 1:** The Baseline Patient Data

Item	Sublobar Resection		Lobectomy		
	No.	Rate (%)	No.	Rate(%)	*P*
**Age groups**					
<40 years	10	8.1	5	2.2	*
40–49 years	20	16.1	20	9.0	
50–59 years	39	31.5	76	34.1	
60–69 years	43	34.7	96	43.0	
70–79 years	12	9.7	26	11.7	
**Marital status**					
Married	122	98.4	221	99.1	0.55
Widowed	2	1.6	2	0.9	
**Educational Backgroung**					
Primary School	49	39.5	88	39.5	0.80
Junior High School	31	25.0	66	29.6	
High School/Technical secondary school	18	14.5	26	11.7	
Associate College	13	10.5	25	11.2	
University	13	10.5	18	9.1	
**Gender**					
Male	15	12.1	99	44.4	*
Female	109	87.9	124	55.6	
**Pathologic Classification**					
Squmous cell cancer	9	7.3	50	22.4	*
Adenocancer	115	92.7	173	77.6	
**TNM Classification**					
IA1	59	47.6	22	9.9	*
IA2	34	27.4	66	29.6	
IA3	6	4.8	46	20.6	
IB	1	0.8	16	7.2	
IIA	0	0.0	8	3.6	
IIB	8	6.5	29	13.0	
IIIA	6	4.8	23	10.3	
IIIB	0	0.0	3	1.3	
IIIC	0	0.0	0	0.0	
IVA	10	8.1	10	4.5	
**Smoking pack–year history**					
Negative	84	67.7	111	49.8	*
Positive	40	32.3	112	50.2	
**Profession**					
Worker	44	35.5	36	16.1	*
Farmer	13	10.5	29	13.0	
Staff	14	11.3	27	12.1	
Civil servant	3	2.4	2	0.9	
Teacher	7	5.6	18	8.1	
OTH	43	34.7	111	49.8	
**Nationality**					
Han Nationality	120	96.8	223	100.0	0.06
Manchu	1	0.8	0	0.0	
Hui Nationality	2	1.6	0	0.0	
Daur	1	0.8	0	0.0	

*P* Sublobar Resection group vs. Lobectomy none group, *: *P*<0.05

**Table 2:** The results of the single factor analysis of PQOL

Factor	Rank Average of 3M PQOL	Me	*p*	Rank Average of 6M PQOL	Me	*p*
**TNM classification**			**			0.09
IA1	152.09	14.00		159.96	6.00	
IA1	170.01	15.00		173.31	7.00	
IA3	181.30	16.00		176.61	7.00	
IB	228.32	21.00		221.32	10.00	
IIA	88.19	11.50		121.44	5.50	
IIB	187.89	16.00		180.09	7.00	
IIIA	2074.10	18.00		188.17	8.00	
IIIB	290.33	27.00		300.50	20.00	
IIIC	\	\		\	\	
IVA	165.05	15.50		157.55	6.00	
**Age groups**			0.16			0.17
<40 years	147.40			139.97		
40-49 years	158.49			168.73		
40-59 years	182.12			183.04		
60-69 years	166.77			164.55		
70-79 years	202.68			200.21		
**Smoking pack-year history**			0.18			0.66
Smokers	182.24	16.00		176.66	7.00	
Nonsmokers	167.57	15.00		171.93	8.00	
**Pathologic classification**			0.07			0.38
Squmous cell cancer	195.36	17.00		184.34	7.00	
Adenocancer	169.62	15.00		171.88	7.50	
**Gender**			0.15			0.63
Male	182.17	16.00		176.76	7.00	
Female	166.67	15.00		171.76	8.00	
**Morpphology**			0.33			0.87
Lung nodules	170.54	15.00		174.59	8.00	
Pulmonary mass	181.86	15.00		12.65	7.00	
**Surgery**			***			*
Lobectomy	193.26	17.00		184.40	8.00	
Sublobar resection	139.37	14.00		155.29	6.00	

Data was reserved for second decimal, *Me* denotes the median, ***: *P*<0.001, **: *P*<0.01, *: *P*<0.05

**Fig 1:**
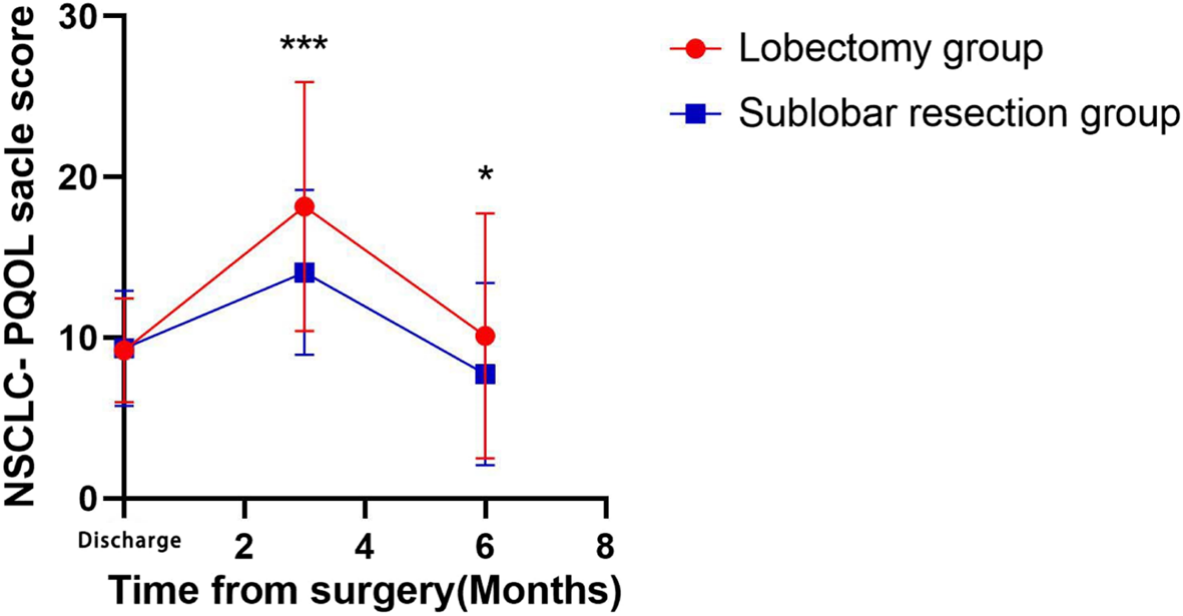
Overall PQOL Scale in NSCLC-PQOL. Overall PQOL was similar at hospital discharge level. At 3 month and 6 months after surgery, there were differences in two groups (*P*<0.05). ***: *P*<0.001, **: *P*<0.01, *: *P*<0.05, ns: no statistic differences

Therefore, we used the surgical method as a categorical variable to explore the pattern of change in the quality of survival in the postoperative period (Fig. 1). There was no significant difference in the quality of survival at the discharge level between the patients in the lobectomy group and the patients in the sublobar resection group. The quality of survival scores of the patients in the two groups increased progressively after discharge suggesting that the quality of survival declined progressively, and the quality of survival of the patients in the sublobar resection group was better than that of the patients in the lobectomy group. The quality of survival gradually improved from 3 months to 6 months postoperatively, and the quality of survival of patients in the lobectomy group was inferior to that of patients in the sublobar resection group, but the difference between the two groups gradually narrowed and approached the discharge level.

**Fig 2:**
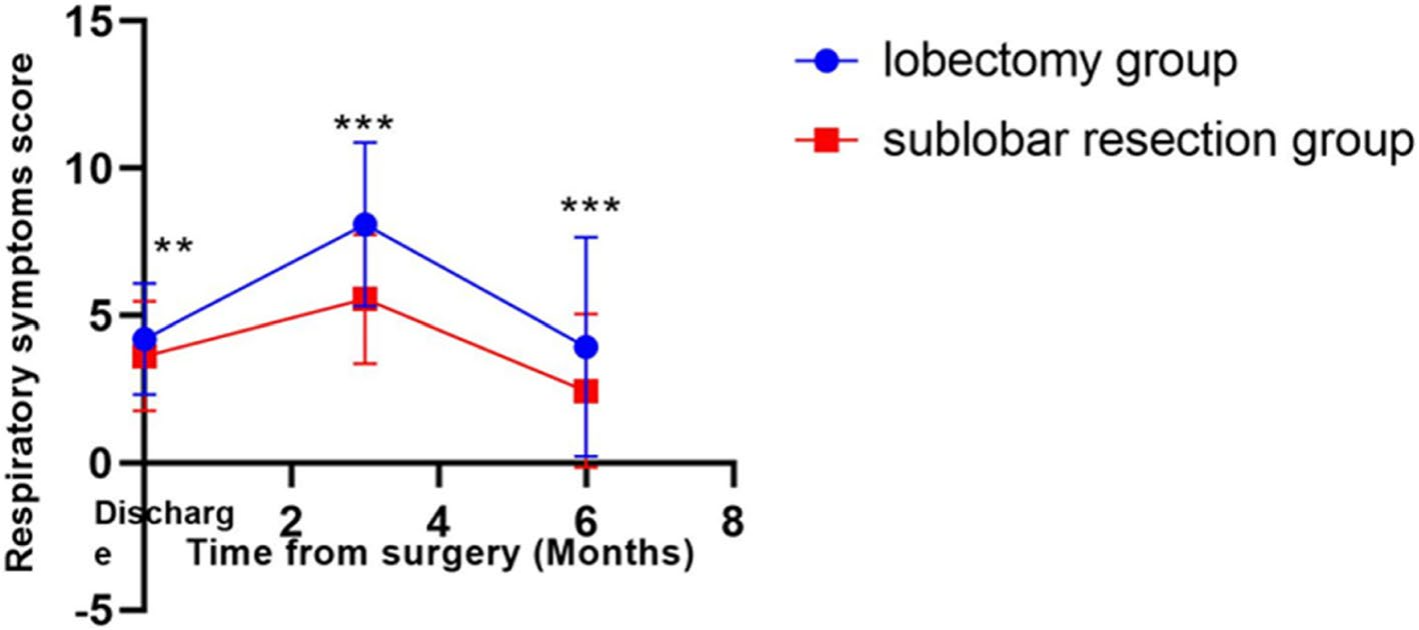
Trends in postoperative respiratory symptom scores. Trends in postoperative respiratory symptom scores are consistent with trends in PQOL changes, Postoperative respiratory symptom scores were smaller in the sublobar resection group than in the lobectomy group at all three time points. (*P*<0.05). ***: *P*<0.001, **: *P*<0.01, *: *P*<0.05, ns: no statistic differences

To investigate how the surgical approach affects postoperative survival quality, this study separately analyzed the effect of the surgical approach on 22 items of survival quality, the results of which can be found in Table 3, it can be seen that the surgical modality mainly affects the symptom subscales, with a major impact on the respiratory score. The respiratory symptom (“breath shortness”, “chest tightness”, “breathlessness”, “cough”, and “expectoration”) scores in the lobectomy group were higher than those in the sublobar resection group at the discharge level, 3 months and 6 months postoperatively (Table 4), and the respiratory scores of the patients in both groups increased gradually from 0 to 3 months postoperatively, declined gradually from 3 to 6 months and approached the level at the time of discharge (Fig. 2), and the pattern of change was in line with the pattern of change in the quality of survival scores. Therefore, the different surgical methods may affect the patients’ postoperative survival quality by influencing the respiratory symptom scores.

**Table 3:** Comparsion of PQOL of two groups

Item	After Surgery (*n*=347)		*P*	Item	After Surgery (*n*=347)		*P*
	lobectomy group	sublobar resection group			lobectomy group	sublobar resection group	
**Breath shortness**				**Chest pain**			
**Discharge**	1.21±0.68	0.51±0.64	***	**Discharge**	0.24±0.52	0.51±0.76	***
**3M**	1.99±0.75	1.12±0.75	***	**3M**	0.43±0.90	0.46±0.76	0.11
**6M**	0.82±0.99	0.44±0.77	***	**6M**	0.27±0.68	0.23±0.54	0.89
**Chest tightness**				**Mental stress**			
**Discharge**	0.86±0.70	0.90±0.79	0.86	**Discharge**	0.33±0.57	0.33±0.54	0.78
**3M**	1.70±0.90	1.06±0.80	***	**3M**	0.51±0.59	0.45±0.70	0.71
**6M**	0.82±1.02	0.48±0.74	0.004 **	**6M**	0.22±0.60	0.07±0.29	0.01*
**Breathlessness**				**I'm disappointed in my struggle with the illness**			
**Discharge**	0.66±0.62	0.60±0.70	0.21	**Discharge**	0.31±0.53	0.25±0.57	0.1
**3M**	1.53±0.88	1.01±0.86	***	**3M**	0.70±0.97	0.31±0.61	***
**6M**	0.81±0.99	0.50±0.77	0.007**	**6M**	0.44±0.64	0.44±0.60	0.84
**Weight loss**				**Irritability**			
**Discharge**	0.49±0.58	0.36±0.56	0.03 *	**Discharge**	0.09±0.31	0.31±0.55	***
**3M**	1.15±0.82	0.49±0.74	***	**3M**	0.70±0.95	0.31±0.38	0.59
**6M**	0.31±0.71	0.24±0.45	0.71	**6M**	0.42±0.66	0.41±0.65	0.9
**Cough**				**I can't accept my illness**			
**Discharge**	0.90±0.69	0.90±0.63	0.84	**Discharge**	0.39±0.60	0.56±0.77	0.09
**3M**	1.61±0.93	1.29±0.67	***	**3M**	0.80±1.11	0.80±1.06	0.9
**6M**	0.87±0.92	0.59±0.77	0.004 **	**6M**	0.50±0.73	0.57±0.80	0.47
**Expectoration**				**Overall-PQOL**			
**Discharge**	0.58±0.63	0.73±0.62	0.03 *	**Discharge**	9.21±3.22	9.35±3.59	0.71
**3M**	1.25±0.94	1.09±0.64	0.12	**3M**	18.17±7.75	14.05±5.13	***
**6M**	0.62±0.68	0.43±0.63	0.005 **	**6M**	10.11±7.63	7.73±5.69	0.02 *
**Pain of the surgical wound**				**I can't take care of myself, such as eating and dressing**			
**Discharge**	0.21±0.44	0.44±0.77	0.02 *	**Discharge**	0.13±0.37	0.13±0.34	0.68
**3M**	0.32±0.67	0.27±0.57	0.72	**3M**	0.12±0.43	0.21±0.55	0.04*
**6M**	0.10±0.30	0.12±0.33	0.52	**6M**	0.08±0.35	0.16±0.39	0.007**

Data was expressed as mean±standard deviation and was reserved for second decimal place; ***: *P*<0.001, **: *P*<0.01, *: *P*<0.05, ns: no statistic differences

**Table 4:** Comparsion of Respiratory symptoms score

Item	After Surgery (*n*=347)		*P*
	lobectomy group	sublobar resection group	
**Respiratory symptoms score**			
**Discharge**	4.21±1.89	3.63±1.85	**
**3M**	8.09±2.78	5.56±2.18	***
**6M**	3.94±3.71	2.44±2.60	***

Data was expressed as mean±standard deviation and was reserved for second decimal place; ***: *P*<0.001, **: *P*<0.01, *: *P*<0.05, ns: no statistic differences

## Discussion

The core aim of anti-tumor treatment is survival. And lobectomy has long been the standard procedure for the surgical treatment of lung cancer. On the one hand, in previous studies, no difference was found between mortality rates after sublobar resection and within 90 days in patients after lobectomy [16]. The time to recurrence (TTR), recurrence–free survival (RFS), and OS showed no difference between those who underwent lobectomy and limited resection (segment or wedge) when adequate numbers of LNs were examined, and the negative surgical margins were confirmed [6, 17]. It has been reported that limited resection is equivalent to lobectomy managing early-stage (T_1–2_ N_0_) NSCLC [3]. And there was no difference in almost all postoperative parameters of intraoperative and postoperative complications between segmentectomy and lobectomy [18]. On the other hand, with the expansion of lung cancer screening, the proportion of patients with early–stage lung cancer is also increasing, and sublobar resection may be the preferred surgical option. The reason for this is that the advantages of sublobar resection include preservation of lung function, low operative morbidity and mortality, low intraoperative blood loss, and short hospital stay [3]. Also, sublobar resection maintains the possibility of curative surgery for secondary primary lung cancer [19, 20]. Against this backdrop, QOL could impact the selection of the specific treatment regimen and monitor response to treatment and disease progression [21–23].

QOL should be an integrated part of the routine clinical visit in oncology practices and is key to the successful interaction between patients and their physicians [24]. Therefore, capturing the patient’s quality of life during treatment and rehabilitation can help to improve patient compliance, thus contributing to our therapeutic philosophy of shrinking the tumor to control and/or relieve symptoms, while trying to prevent QOL deterioration.

As the evaluation of QOL is a subjective and individual abstract concept that depends on a person’s circumstances [15]. We developed the powerful and quick NSCLC–PQOL scale to quantify the multidimensional quality of survival. With the use of low-dose spiral CT, the age range of lung cancer patients is gradually increasing, the age of the population included in this study ranged from 30 to 70 years old. A recent study has revealed that the Long–term survival rate has no significantly difference between 70 years old NSCLC patients and the younger ones [25, 26]. Therefore, there is no selection bias for different age groups in the creation of the scale. It is also worth noting that the NSCLC–PQOL scale has maximized the inclusion of multidimensional quality of survival for patients due to the fact that lung cancer patients are older and more often retired, and the importance of considering the impact of the disease and treatment on the patient’s professional life and finances at any point in the treatment process has not been considered in this study in terms of the quality of survival examined. Considering the scale with fewer items shows better responsiveness during the clinical studies [27], the NSCLC–PQOL scale was designed to be responsive and comprehensive. NSCLC–PQOL scale to become the appraisal tool for NSCLC patients who underwent surgery.

In this study, we analyzed the characteristics of the short-term quality of survival of non-small cell lung cancer patients in the 6 months after surgery and its changing pattern, and the quality of survival of patients after sublobar resection was better than that of the lobectomy group at several time points. We also found that the postoperative quality of life of patients in both groups gradually deteriorated from the discharge to 3 months postoperatively, and then gradually improved between 3 and 6 months postoperatively. The results of this study corroborate the findings of related studies that patients undergoing lobectomy have a poorer quality of survival related to somatic symptoms at 3 months postoperatively [28] . Although we were unable to derive the time when the quality of life scores reached the peak due to the small number of data collection points, the gradual recovery of the quality of life 6 months postoperatively is consistent with the previous studies. This is consistent with the pattern of change in postoperative physical symptoms such as cough and shortness of breath in lung cancer patients in other studies [29, 30]. Therefore, combining the results of related studies in which the extent of surgical resection was a strong predictor of patients’ QOL beyond 6 months postoperatively [31], we hypothesise that the differences in surgical approach, is also a major factor affecting the quality of patients’ survival within the 6 months postoperatively.

In this study, we found that the difference in surgical methods mainly affected the respiratory symptoms leading to differences in the quality of survival between the two groups. In this study Furthermore, we observed that the respiratory symptoms of patients in the sublobar resection group were better than those of patients in the lobectomy group at all three postoperative time points and that the rate and degree of deterioration of respiratory symptoms in the sublobar resection group was less than that in the lobectomy group after surgery. According to the results of the most recent clinical trial, patients in the sublobar resection group had better lung function than those in the lobectomy group at 6 months postoperatively, but there was no difference at 1 year postoperatively [16]. Such a difference may be related to the ability of sublobar resection to be more preservative of lung function. Previous studies reported better preservation of pulmonary function after limited resection than after lobectomy during the initial postoperative period, with this difference decreasing over time [32–34]. We also found that the difference in respiratory symptom scores between the two groups at 6 months postoperatively was smaller than that at 3 months postoperatively (Table 4), further corroborating the above studies. Therefore sublobar resection is not a preferred treatment option while ensuring complete tumor resection.

In this study, we observed no significant difference in the psycho-social and social-life quality of life scores between patients in the lobectomy group and those in the sublobectomy group. Meanwhile, we found the PQOL of sublobar resection groups was not significantly better than the lobectomy group in spiritual–psychological and social life domains. Thus, the sublobar resection group self-reported better overall PQOL than lobectomy 3 and 6 months after surgery. It showed that less extent of surgical resection (ESR) indicated better PQOL to ensure therapeutic gains [35]. According to a recent study about disease-free survival (DFS) of lung cancer patients, there was no difference in global, physical, psychosocial, medical interaction, marital, and sexual QOL variables between short–versus long–term lung cancer survivors at an average of 3.4 years after diagnosis [36]. This finding may suggest that surgical procedures will not influence the PQOL on the above categories. This phenomenon may be related to the mental and life–affecting effects of the disease itself, regardless of which treatment the patient has received.

This study also has some limitations. First, as a single–center study, the samples were under–represented. In future studies, we hope to conduct a multicenter trial to increase the universality. Second, as there has been no gold standard for assessing the PQOL of NSCLC patients, we could not evaluate the criterion-related validity of the NSCLC–PQOL scale. Third, we hope to carry out a longitudinal study about PQOL as this study is cross-sectional. Fourth, we were unable to assess for recurrence and survival for all patients because of the short follow–up time.

## Conclusion

1. The surgical approach may affect the quality of postoperative survival by influencing postoperative respiratory symptoms, and the quality of postoperative survival was better in patients undergoing sublobar resection than in those undergoing lobectomy at 3 months as well as at 6 months postoperatively.
2. NSCLC–PQOL scale exhibited satisfactory reliability, validity and feasibility, which is a useful evaluation tool.

## Innovation

1. This study describes the pattern of change and differences in postoperative multidimensional quality of survival in NSCLC patients undergoing lobectomy and those undergoing sublobar resection.
2. We developed the NSCLC–PQOL scale to balance comprehensiveness and feasibility.
3. We used stepwise multiple linear regression analyses as well as other statistical methods to screen entries of the scale.

### Supplementary Information

**Additional file 1: Supplementary Table 1.** Statistical results of Spearman correlation coefficient method. **Supplementary Table 2.** Statistical results of item distribution. **Supplementary Table 3.** Statistics of scale data description. **Supplementary Table 4.** Analysis of multiple stepwise linear regression. **Supplementary Table 5.** Analysis of Cronbach's α coefficient. **Supplementary Table 6.** Analysis of Cronbach's α coefficient. **Supplementary Table 7.** Correlation coefficient between each subscale and the total scale. **Supplementary Table 8.** Primary NSCLC–PQOL Scale. **Supplementary Table 9.** NSCLC–PQOL Scale. Development of the scale [37].

## Data Availability

The datasets used and/or analysed during the current study are available from the corresponding author on reasonable request.

## Abbreviations

PQOL: Postoperative quality of life
NSCLC: Non–small–cell lung cancer
NSCLC–PQOL: Quality of life scale of non–small–cell lung cancer
GGO: Ground glass opacity
OS: Overall survival
LNs: lymph nodes
FACT–L: Functional assessment of cancer therapy–lung
LCSS: Lung cancer symptom scale
SF–36 Health Survey: Short form 36 health survey questionnaire
QOL: Quality of Life
ERG: Experts Reference Group
LDCT: Low–dose computed tomography transmission scan
TTR: Time to recurrence
RFS: Recurrence–free survival
PFTs: Pulmonary function tests
ESR: Extent of surgical resection
DFS: Disease–free survival
