# Supplementary material for: Quality of life after lung cancer surgery: sublobar resection versus lobectomy
Source: BMC Surg. 2023 Nov 18;23:353. doi: 10.1186/s12893-023-02259-1 (PMC10657598; doi:10.1186/s12893-023-02259-1)
Supplement: Supplementary file 1 — Additional file 1: Supplementary Table 1. Statistical results of Spearman correlation coefficient method. Supplementary Table 2. Statistical results of item distribution. Supplementary Table 3. Statistics of scale data description. Supplementary Table 4. Analysis of multiple stepwise linear regression. Supplementary Table 5. Analysis of Cronbach's α coefficient. Supplementary Table 6. Analysis of Cronbach's α coefficient. Supplementary Table 7. Correlation coefficient between each subscale and the total scale. Supplementary Table 8. Primary NSCLC–PQOL Scale. Supplementary Table 9. NSCLC–PQOL Scale. Development of the scale [37]. [file 12893_2023_2259_MOESM1_ESM.zip › Revised supplementary material.docx]

**Development of the scale**

***Forming alternative items and initial scales.*** Through literature research, case reviews, collecting opinions of patients, and soliciting the personal experiences of experts, the research group collected alternative items. Then the core group formed the initial scale, consisting of three subscales with 25 items. There were 14 items in the signs and symptoms subscale (scale A), 7 items in the psychological and psychiatric subscale (scale B) and 4 items in social–life subscale (scale C) (Supplementary Table 8). The score of each item ranges from 0 to 3, indicating that the severity of related symptoms or conditions is from none to extremely serious. The total score refers to the sum of the scores for all items, and the subscale score is the sum of the scores for each subscale item.

***Preliminary investigation.*** During the pilot survey, three NSCLC patients and 10 healthy people were investigated with the primary NSCLC–PQOL scale. Results showed that no item was ambiguous, added or deleted.

***Developing the final scale.* (1) Demographic characteristics of the study subjects:** The subjects of this study are 347 patients who received operation and treatment in the Department of Lung Cancer Surgery at Tianjin Medical University Cancer Institute and Hospital from November 2019 to May 2021, and then we used primary NSCLC–PQOL scale to evaluate the severity of the patient’s signs and symptoms, social adaptation and mental well‐being at hospital discharge level, three months and six months after their surgery. The data from six months after surgery was used to develop the NSCLC-PQOL scale. The demographic characteristics are shown in Table 1. (**2) Screening scale items by** **correlation coefficient method:** The correlation coefficient method is to calculate the correlation coefficient between each item and the secondary variable (the subscale) to which the item belongs and then deletes the items with the absolute value of the correlation coefficient less than 0.4. The data was calculated by the Spearman test, and the items which could be eliminated are shown in Supplementary Table 1. (**3) Analysis of item distribution inspection method:** The method inspects the distinguishability of scale items from the central tendency of item answers’ distribution. Generally, the rate of item choices over 80% can be deleted indicating the poor specificity of the items. The items proposed to be deleted shown in Supplementary Table 2. (**4) Analysis of discrete tendency: T**he higher the degree of dispersion, the stronger its ability to distinguish different symptoms shows. Items with standard deviation less than 0.7 should be deleted. The results items that could be excluded shown in Supplementary Table 3. (**5****) Analysis of multiple stepwise linear regression:** This method takes the original items in the scale as independent variables and the total score as dependent variables. Then we used different independent variables to estimate the dependent variables. It can calculate the contribution of independent variables to the dependent variables. It is considered to include the items with P < 0.05 in the equation. According to multiple stepwise linear regression analysis results (Supplementary Table 4), no item should be deleted from the scale. (**6) Eliminating items by** **Cronbach's α coefficient and examining the scale:** We respectively calculated Cronbach's α coefficient of each item and the total scale. The overall Cronbach's α coefficient of the previous total scale is 0.837. After the removal of a certain item, we compared the recalculated Cronbach's α coefficient of the total scale and the previous one. If the recalculated value of the scale increases after removing an item, the item should be deleted. The results are shown in Supplementary Table 5.

In combination with the above steps, it was considered that items which met three or more of the five screening methods should be deleted. As pain is an important part of the evaluation of PQOL, relevant items were retained after consulting the experts reference group. Finally, “hair loss”, “vomiting”, and “difficulty in remembering” would be deleted to form the final scale (Supplementary Table 9).

**Evaluation of the final scale**

***Feasibility.*** The clinical questionnaire’s return effective response rates were both 97.5%. All the questionnaires returned were filled without missing items. The vast majority of patients understood the contents of each item. The mean filling time was 11.25±2.54 minutes, showing the patients accepted the scale well.

***Reliability evaluation.*** This study adopted the reliability evaluation of calculating Cronbach's α coefficient. The Cronbach's α coefficient of the total scale, the signs and symptoms subscale, the Psychological and Psychiatric subscale and the social life subscale were 0.84, 0.74, 0.75 and 0.84 (Supplementary Table 6). All the coefficients were greater than 0.7 ^(37)^. Thus, we can conclude that the scale’s internal consistency was good.

The Spearman-Brown coefficient of the total scale, the signs and symptoms domain, Psychological and Psychiatric Scale and social life scale were 0.60, 0.60, 0.71 and 0.86 (Supplementary Table 6). The coefficients obtained are all less than 0.9, and the Spearman-Brown coefficients of the signs and symptoms and the total scale were close to 0.7, the total scale and the cross–item consistency of each dimension were acceptable, and the contents reflected by the scale were relatively the same and stable.

In conclusion, we can conclude that the reliability of the NSCLC–PQOL scale was good.

***Validity.* (1)** **Content validity:** The development process was based on the international QOL scale development principles. The item pool of the scale was created by combining the medical records reviews, the literature analysis, validated scales and other sources. Then the items were screened at multiple levels to form the final scale, so the NSCLC–PQOL scale had good content validity. (**2) Structural validity:** The Spearman coefficient was calculated to determine the correlation between the subscale and the total scale (Supplementary Table 7). The correlation coefficients between each subscale and the total scale were over 0.4, which showed that the subscales have a good correlation with the total scale and the contents of the scales were independent of each other but coming from the same unity.
