# Supplementary material for: Quality of life after lung cancer surgery: sublobar resection versus lobectomy
Source: BMC Surg. 2023 Nov 18;23:353. doi: 10.1186/s12893-023-02259-1 (PMC10657598; doi:10.1186/s12893-023-02259-1)
Supplement: Supplementary file 1 — Additional file 1: Supplementary Table 1. Statistical results of Spearman correlation coefficient method. Supplementary Table 2. Statistical results of item distribution. Supplementary Table 3. Statistics of scale data description. Supplementary Table 4. Analysis of multiple stepwise linear regression. Supplementary Table 5. Analysis of Cronbach's α coefficient. Supplementary Table 6. Analysis of Cronbach's α coefficient. Supplementary Table 7. Correlation coefficient between each subscale and the total scale. Supplementary Table 8. Primary NSCLC–PQOL Scale. Supplementary Table 9. NSCLC–PQOL Scale. Development of the scale [37]. [file 12893_2023_2259_MOESM1_ESM.zip › supplementary table.docx]

| **Supplementary Table 1. Statistical results of Spearman correlation coefficient method** | | | |
| --- | --- | --- | --- |
| **[Item](javascript:;)** | **Correlation coefficient** | **[Item](javascript:;)** | **Correlation coefficient** |
| Breath shortness | 0.7 | Difficulty in remembering ^🞲^ | 0.2^🞲^ |
| Chest tightness | 0.7 | Mental stress | 0.4 |
| Breathlessness | 0.7 | Difficulty in concentrating (reading books and newspapers, etc.) | 0.8 |
| Weight loss | 0.3^🞲^ | I'm disappointed in my struggle with the illness | 0.8 |
| Cough | 0.5 | I feel very depressed | 0.6 |
| Expectoration | 0.5 | Irritability | 0.7 |
| Hair loss^🞲^ | 0.3^🞲^ | The illness interferes with my social activities. | 0.8 |
| Poor appetite | 0.4 | I can't do my previous leisure activities | 0.9 |
| Pain of the surgical wound^🞲^ | 0.1^🞲^ | I can't accept my illness | 0.7 |
| Chest pain^🞲^ | 0.2^🞲^ | I can't take care of myself, such as eating and dressing | 0.60 |
| Other pain^🞲^ | 0.2^🞲^ | The illness interferes with my family life | 0.90 |
| Vomiting^🞲^ | 0.2^🞲^ |  |  |
| Fatigue | 0.6 |  |  |
| Insomnia^🞲^ | 0.3^🞲^ |  |  |
| **Notes:** ^🞲^ ,Items to be deleted with Correlation coefficient less than 0.4 | | | |

| **Supplementary Table 2.** Statistical results of item distribution | | | | |
| --- | --- | --- | --- | --- |
| **Item** | Frequency of option | | | |
|  | 0 | 1 | 2 | 3 |
| Breath shortness | 0.54 | 0.30 | 0.09 | 0.07 |
| Chest tightness | 0.50 | 0.31 | 0.11 | 0.07 |
| Breathlessness | 0.51 | 0.32 | 0.10 | 0.07 |
| Weight loss | 0.79 | 0.15 | 0.04 | 0.02 |
| Cough | 0.46 | 0.40 | 0.08 | 0.08 |
| Expectoration | 0.54 | 0.37 | 0.10 | 0.00 |
| Hair loss^🞲^ | 0.82^🞲^ | 0.10 | 0.05 | 0.02 |
| Poor appetite^🞲^ | 0.84^🞲^ | 0.12 | 0.03 | 0.01 |
| Pain of the surgical wound^🞲^ | 0.89^🞲^ | 0.11 | 0.00 | 0.00 |
| Chest pain | 0.83^🞲^ | 0.10 | 0.06 | 0.01 |
| Other pain^🞲^ | 0.81^🞲^ | 0.15 | 0.03 | 0.01 |
| Vomiting^🞲^ | 0.93^🞲^ | 0.05 | 0.01 | 0.01 |
| Fatigue | 0.61 | 0.27 | 0.10 | 0.03 |
| Insomnia | 0.67 | 0.20 | 0.07 | 0.06 |
| Difficulty in remembering ^🞲^ | 0.93^🞲^ | 0.05 | 0.01 | 0.01 |
| Mental stress^🞲^ | 0.88^🞲^ | 0.10 | 0.01 | 0.02 |
| Difficulty in concentrating (reading books and newspapers, etc.) | 0.59 | 0.28 | 0.11 | 0.02 |
| I'm disappointed in my struggle with the illness | 0.63 | 0.31 | 0.06 | 0.01 |
| I feel very depressed | 0.71 | 0.22 | 0.07 | 0.00 |
| The illness interferes with my family life^🞲^ | 0.81^🞲^ | 0.16 | 0.03 | 0.00 |
| The illness interferes with my social activities^🞲^ | 0.84^🞲^ | 0.14 | 0.02 | 0.00 |
| I can't do my previous leisure activities^🞲^ | 0.81^🞲^ | 0.16 | 0.03 | 0.00 |
| I can't accept my illness | 0.62 | 0.26 | 0.11 | 0.02 |
| I can't take care of myself, such as eating and dressing^🞲^ | 0.91^🞲^ | 0.08 | 0.01 | 0.00 |
| Irritability | 0.67 | 0.26 | 0.07 | 0.01 |
| Notes:^🞲^, Items to be deleted with frequency of option greater than 0.8 | | | | |

| **Supplementary Table 3. Statistics of scale data description** | | | |
| --- | --- | --- | --- |
| [Item](javascript:;) | Standard deviation | [Item](javascript:;) | Standard deviation |
| Breath shortness | 0.90 | Difficulty in remembering ^🞲^ | 0.46^🞲^ |
| Chest tightness | 0.92 | Mental stress^🞲^ | 0.52^🞲^ |
| Breathlessness | 0.91 | Difficulty in concentrating (reading books and newspapers, etc.) | 0.77 |
| Weight loss^🞲^ | 0.63^🞲^ | I'm disappointed in my struggle with the illness^🞲^ | 0.63^🞲^ |
| Cough | 0.88 | I feel very depressed^🞲^ | 0.62^🞲^ |
| Expectoration^🞲^ | 0.67^🞲^ | Irritability^🞲^ | 0.66^🞲^ |
| Hair loss^🞲^ | 0.65^🞲^ | The illness interferes with my social activities^🞲^ | 0.43^🞲^ |
| Poor appetite^🞲^ | 0.54^🞲^ | I can't do my previous leisure activities^🞲^ | 0.48^🞲^ |
| Pain of the surgical wound^🞲^ | 0.31^🞲^ | I can't accept my illness | 0.75 |
| Chest pain^🞲^ | 0.63^🞲^ | I can't take care of myself , such as eating and dressing^🞲^ | 0.36^🞲^ |
| Other pain^🞲^ | 0.55^🞲^ | The illness interferes with my family life^🞲^ | 0.48^🞲^ |
| Vomiting^🞲^ | 0.40^🞲^ |  |  |
| Fatigue | 0.78 |  |  |
| Insomnia | 0.88 |  |  |
| Notes: ^🞲^, Items to be deleted with standard deviation less than 0.7 | | | |

| **Supplementary Table 4. Analysis of multiple stepwise linear regression** | | |
| --- | --- | --- |
| **Item** | standardized coefficient | ***P*** |
| Breathlessness | 0.128 | ^🞲🞲🞲^ |
| Mental stress | 0.075 | ^🞲🞲🞲^ |
| The illness interferes with my family life | 0.068 | ^🞲🞲🞲^ |
| Cough | 0.124 | ^🞲🞲🞲^ |
| Insomnia | 0.123 | ^🞲🞲🞲^ |
| I’m disappointed in my struggle with the illness | 0.090 | ^🞲🞲🞲^ |
| I can’t accept my illness | 0.108 | ^🞲🞲🞲^ |
| Weight loss | 0.090 | ^🞲🞲🞲^ |
| Fatigue | 0.112 | ^🞲🞲🞲^ |
| Chest pain | 0.091 | ^🞲🞲🞲^ |
| I feel very depressed | 0.089 | ^🞲🞲🞲^ |
| Hair loss | 0.093 | ^🞲🞲🞲^ |
| Difficulty in concentrating (reading books and newspapers, etc.) | 0.110 | ^🞲🞲🞲^ |
| Breath shortness | 0.131 | ^🞲🞲🞲^ |
| Expectoration | 0.095 | ^🞲🞲🞲^ |
| Other pain | 0.079 | ^🞲🞲🞲^ |
| Poor appetite | 0.077 | ^🞲🞲🞲^ |
| I can’t do my previous leisure activities | 0.068 | ^🞲🞲🞲^ |
| Vomiting | 0.057 | ^🞲🞲🞲^ |
| Pain of the surgical wound | 0.044 | ^🞲🞲🞲^ |
| The illness interferes with my social activities | 0.062 | ^🞲🞲🞲^ |
| Difficulty in remembering | 0.066 | ^🞲🞲🞲^ |
| Chest tightness | 0.131 | ^🞲🞲🞲^ |
| I can’t take care of myself, such as eating and dressing | 0.052 | ^🞲🞲🞲^ |
| Irritability | 0.094 | ^🞲🞲🞲^ |
| Notes: ^🞲🞲🞲^: *P*<0.001 | | |

| **Supplementary Table 5.** Analysis of Cronbach's α coefficient | | | |
| --- | --- | --- | --- |
| [Item](javascript:;) | Cronbach's α after deleting the item | [Item](javascript:;) | Cronbach's α after deleting the item |
| Breath shortness | 0.819 | Difficulty in remembering ^🞲^ | 0.839 ^🞲^ |
| Chest tightness | 0.819 | Mental stress | 0.825 |
| Breathlessness | 0.818 | Difficulty in concentrating (reading books and newspapers, etc.) | 0.831 |
| Weight loss | 0.833 | I'm disappointed in my struggle with the illness | 0.827 |
| Cough | 0.833 | I feel very depressed | 0.827 |
| Expectoration | 0.835 | Irritability | 0.827 |
| Hair loss | 0.835 | The illness interferes with my social activities | 0.832 |
| Poor appetite | 0.832 | I can't do my previous leisure activities | 0.831 |
| Pain of the surgical wound ^🞲^ | 0.839 ^🞲^ | I can't accept my illness | 0.831 |
| Chest pain ^🞲^ | 0.840 ^🞲^ | I can't take care of myself, such as eating and dressing | 0.832 |
| Other pain ^🞲^ | 0.839 ^🞲^ | The illness interferes with my family life | 0.830 |
| Vomiting ^🞲^ | 0.838 ^🞲^ |  |  |
| Fatigue | 0.828 |  |  |
| Insomnia ^🞲^ | 0.842 ^🞲^ |  |  |
| Notes: ^🞲^: The overall Cronbach's α coefficient increased after the entry is deleted, | | | |

| **Supplementary Table 6.** Analysis of Cronbach's α coefficient | | |
| --- | --- | --- |
| Item | Cronbach's α coefficient | Spearman–Brown coefficient |
| Total Scale | 0.81 | 0.60 |
| Signs and Symptoms Domain | 0.74 | 0.60 |
| Psychological and Psychiatric Domain | 0.75 | 0.71 |
| Social Life Domain | 0.84 | 0.86 |

| **Supplementary Table 7.** Correlation coefficient between each subscale and the total scale | |
| --- | --- |
| **Subscale** | **Total scale** |
| Signs and symptoms scale | 0.85 |
| Psychological and Psychiatric scale | 0.70 |
| Social life scale | 0.45 |

| **Supplementary Table 8.** Primary NSCLC–PQOL Scale | | | |
| --- | --- | --- | --- |
| **Number** | **Item** | **Number** | **Item** |
| A1 | Breath shortness | B1 | Difficulty in remembering |
| A2 | Chest tightness | B2 | Mental stress |
| A3 | Breathlessness | B3 | Difficulty in concentrating (reading books and newspapers, etc.) |
| A4 | Weight reduction | B4 | I'm disappointed in my struggle with the illness |
| A5 | Cough | B5 | I feel very depressed |
| A6 | Expectoration | B6 | Irritability |
| A7 | Hair loss | B7 | I can't accept my illness |
| A8 | Appetite loss | C1 | The illness interferes with my social activities |
| A9 | Pain of the surgical wound | C2 | I can't do my previous leisure activities |
| A10 | Chest pain | C3 | I can't take care of myself, such as eating and dressing |
| A11 | Other pain | C4 | ·       The illness interferes with my domesticity |
| A12 | Vomiting | ––– | ––– |
| A13 | Fatigue | ––– | ––– |
| A14 | Insomnia | ––– | ––– |
| Note: Item A1: shortness of breath is more severe than those of the general population; Item A2: discomfort in chest but no pain; Item A3: breathing with a sense of suffocation; Item A4: no observed weight loss—0-point, 0~5–kg weight loss—1 point, 5~10–kg weight loss—2 point, more than 10Kg weight loss—3 point; Item C1: domesticity means functional interaction between family members | | | |

| **Supplementary Table 9.** NSCLC–PQOL Scale | | | |
| --- | --- | --- | --- |
| **Number** | **Item** | **Number** | **Item** |
| A1 | Breath shortness | B1 | Mental stress |
| A2 | Chest tightness | B2 | Difficulty in concentrating (reading books and newspapers, etc.) |
| A3 | Breathlessness | B3 | I'm disappointed in my struggle with the illness |
| A4 | Weight reduction | B4 | I feel very depressed |
| A5 | Cough | B5 | Irritability |
| A6 | Expectoration | B6 | I can't accept my illness |
| A7 | Appetite loss | C1 | The illness interferes with my social activities |
| A8 | Pain of the surgical wound | C2 | I can't do my previous leisure activities |
| A9 | Chest pain | C3 | I can't take care of myself, such as eating and dressing |
| A10 | Other pain | C4 | The illness interferes with my domesticity |
| A11 | Fatigue |  |  |
| A12 | Insomnia |  |  |
| Note: Item A1: shortness of breath is more severe than those of the general population; Item A2: discomfort in chest but no pain; Item A3: breathing with a sense of suffocation; Item A4: no observed weight loss—0-point, 0~5–kg weight loss—1 point, 5~10–kg weight loss—2 point, more than 10Kg weight loss—3 point; Item C1: domesticity means functional interaction between family members | | | |
